# Application of an Ovate Leaf Shape Model to Evaluate Leaf Bilateral Asymmetry and Calculate Lamina Centroid Location

**DOI:** 10.3389/fpls.2021.822907

**Published:** 2022-01-17

**Authors:** Yirong Li, Yiwen Zheng, David A. Ratkowsky, Hailin Wei, Peijian Shi

**Affiliations:** ^1^Bamboo Research Institution, College of Science, Nanjing Forestry University, Nanjing, China; ^2^Tasmanian Institute of Agriculture, University of Tasmania, Hobart, TAS, Australia; ^3^Hunan Academy of Forestry, Changsha, China

**Keywords:** centroid ratio, lamina area, leaf petiole, Lobry-Rosso-Flandrois equation, model validity

## Abstract

Leaf shape is an important leaf trait, with ovate leaves common in many floras. Recently, a new leaf shape model (referred to as the MLRF equation) derived from temperature-dependent bacterial growth was proposed and demonstrated to be valid in describing leaf boundaries of many species with ovate leaf shape. The MLRF model’s parameters can provide valuable information of leaf shape, including the ratio of lamina width to length and the lamina centroid location on the lamina length axis. However, the model wasn’t tested on a large sample of a single species, thereby limiting its overall evaluation for describing leaf boundaries, for evaluating lamina bilateral asymmetry and for calculating lamina centroid location. In this study, we further test the model using data from two Lauraceae species, *Cinnamomum camphora* and *Machilus leptophylla*, with >290 leaves for each species. The equation was found to be credible for describing those shapes, with all adjusted root-mean-square errors (RMSE) smaller than 0.05, indicating that the mean absolute deviation is smaller than 5% of the radius of an assumed circle whose area equals lamina area. It was also found that the larger the extent of lamina asymmetry, the larger the adjusted RMSE, with approximately 50% of unexplained variation by the model accounted for by the lamina asymmetry, implying that this model can help to quantify the leaf bilateral asymmetry in future studies. In addition, there was a significant difference between the two species in their centroid ratio, i.e., the distance from leaf petiole to the point on the lamina length axis associated with leaf maximum width to the leaf maximum length. It was found that a higher centroid ratio does not necessarily lead to a greater investment of mass to leaf petiole relative to lamina, which might depend on the petiole pattern.

## Introduction

A leaf of a woody plant usually consists of a lamina, a petiole (or a pseudo-petiole) and a sheath. In the literature, leaf shape often refers just to the lamina shape and does not involve the morphological characteristics of the leaf petiole. As an important photosynthetic organ of plants, the leaf has always been a research hotspot, and leaf traits including lamina size (mass and area), lamina thickness, leaf shape, and lamina vein patterns are widely studied because those measures are intimately associated with the responses of plants to climate and environmental stress (Wright et al., 2004, 2017; Chitwood and Sinha, 2016; Baird et al., 2021). The leaf is crucial to the growth and development of plants, with the characteristics and variation of leaf structure directly affecting absorption and utilization of light energy and nutrients (Smith et al., 1997; Daas-Ghrib et al., 2011). Previous studies have shown that there is a tradeoff between the photosynthetic returns from increasing lamina area and the investment in leaf physical support and hydraulic systems from increasing lamina mass (Niklas et al., 2007; Huang et al., 2019a,b, 2020; Guo et al., 2021). Lamina thickness and leaf shape have been demonstrated to affect such a tradeoff (Niinemets et al., 2007; Lin et al., 2018, 2020). There is a large variation in leaf shape among different species and conspecfics, and it is often used to assist in identifying and classifying plants. Leaf shape is controlled by genetic, physiological, and ecological factors (Nicotra et al., 2011). Leaf shape and leaf venation pattern are closely related, and interact with each other in formation (Dengler and Kang, 2001; Runions et al., 2017). The ratio of lamina width to lamina length is usually used as the leaf shape indicator (Lin et al., 2020). The ovate leaf shape is common in many floras. The centroids of ovate leaves are closer to the lamina base than those of elliptical and obovate leaves, and thus the support costs of petioles for ovate leaves tend to be lower (Niinemets et al., 2007).

Lamina bilateral symmetry can be regarded as one of the leaf shape features (Shi et al., 2020a). How to measure the bilateral symmetry of the lamina is an important scientific issue. The standardized index (SI) was proposed to quantify the extent of lamina bilateral asymmetry based on the relative area differences of different sub-regions between both sides of the lamina (Shi et al., 2018a). The heterogeneity of light in the tree crown contributes to lamina bilateral asymmetry to a great degree (Wang et al., 2018; Guo et al., 2020). The two sides of some laminas might expose light in an irregular pattern because of the architectural structure of trees. Relative to leaf length, leaf width has a smaller variation for broad-leaved plants, especially those with hierarchical reticulate leaf venation (Shi et al., 2018b). For many plants, the bilateral symmetry is often slightly influenced by a skewed lamina apex. Wang et al. (2020) verified that a skewed lamina apex is likely to be beneficial to drainage on the lamina surface.

**FIGURE 1:**
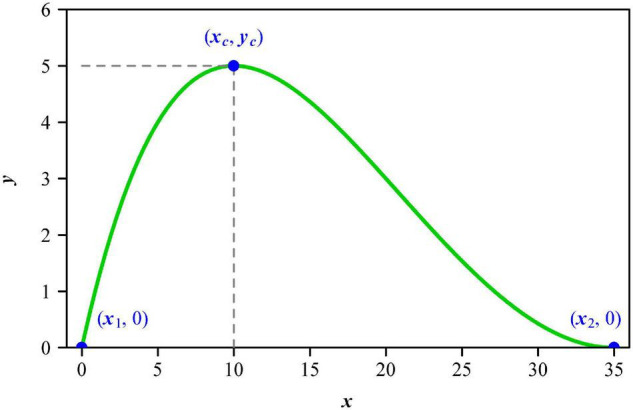
The curve generated by the modified Lobry-Rosso-Flandrois (MLRF) equation, which represents a side of a lamina. Here, *x*_1_ and *x*_2_ represent the lamina base and lamina apex, respectively; *x*_c_ represents the point on the lamina length axis associated with the lamina maximum width 2*y*_c_; δ controls the curvature of this curve. In this study, the location of lamina base (i.e., *x*_1_) is fixed to be 0. This means that the MLRF equation only has four parameters: *y*_c_, *x*_c_, *x*_2_, and δ.

It is valuable to construct a parametric model to describe leaf shape. There are many models for calculating leaf size based on leaf length and width, using these two one-dimensional measures to reflect the influence of leaf shape on the calculation of leaf size (Dornbusch et al., 2011; Shi et al., 2015, 2019; Yu et al., 2020; Schrader et al., 2021). The principle of similarity suggests that an object’s area is proportional to the square of its length (Thompson, 1917); however, the validity of this principle is demonstrated to be largely affected by the variation in the ratio of lamina width to length (Shi et al., 2019; Yu et al., 2020). In other words, the square relationship between lamina area and lamina length depends on the variation in leaf shape. There are linear, lanceolate, and elliptical leaf shape models (Dornbusch et al., 2011; Shi et al., 2015; Li et al., 2021). Although the ovate leaf shape is very common in many floras, this leaf shape was mathematically modeled only recently (Shi et al., 2021). Dornbusch et al. (2011) proposed a step model to describe the linear and lanceolate leaf shape of plants, especially crops, but this model could not produce a round lamina base. Shi et al. (2015) developed a simplified Gielis equation based on its original version (Gielis, 2003) to describe the shape of bamboo leaves. The validity of the simplified Gielis equation has been confirmed using the leaves of 42 bamboo species (Lin et al., 2016). Nevertheless, the simplified Gielis equation cannot produce concave curves close to the lamina apex that a typical ovate leaf shape usually has (Shi et al., 2021). The original Lobry-Rosso-Flandrois (LRF) equation was used to describe the relationship between developmental (or growth) rate and temperature of poikilotherms and microbes (Lobry et al., 1991; Rosso et al., 1993; Ratkowsky and Reddy, 2017). To increase the flexibility of curve fitting, Shi et al. (2017) proposed the modified LRF equation (referred to as MLRF model hereafter for convenience) by adding a parameter δ, which was then able to describe actual ovate leaf shapes (Shi et al., 2021). The resulting modified leaf shape equation has four parameters, all with geometrical meanings: the first one is half lamina maximum width (*y*_*c*_); the second one is the distance from lamina base to a point on the lamina length axis associated with leaf maximum width (*x*_*c*_); the third one is lamina length (*x*_2_); the last one (i.e., δ) controls the curvature of a curve (Figure 1). These parameters can be potentially applied to reflect leaf shape, e.g., the quotient of *x*_*c*_ and *x*_2_, which is referred to as the centroid ratio, can reflect the location of the lamina centroid on the lamina length axis for an ovate or obovate leaf shape. If the quotient is smaller than 0.5, this denotes that the lamina centroid is closer to the lamina base (i.e., an ovate leaf shape); if the quotient is larger than 0.5, this means that the lamina centroid is closer to the lamina apex (i.e., an obovate leaf shape); if the quotient is equal or approximate to 0.5, this indicates that the lamina centroid tends to be located at the midpoint of the lamina length. However, the influence of the location of the lamina centroid on the allocation of mass between the leaf petiole and the leaf lamina is unknown. The MLRF model provides an approach for examining whether the lamina centroid ratio can affect the ratio of leaf petiole mass to lamina mass. It is mistaken to directly use the maximum distance between two points on the lamina edge as lamina length, because lamina bilateral asymmetry can lead to an inaccuracy using such an approach. The estimate of the parameter *x*_2_ is better as a candidate leaf length, because lamina apex might largely deviate from the midvein axis for some leaves, leading to an over-estimation or under-estimation for the lamina length defined from lamina base to lamina apex (Schrader et al., 2021). The MLRF equation predicts perfectly a bilateral symmetrical leaf shape, so the theoretical lamina length (*x*_2_) is on the midvein length.

**FIGURE 2:**
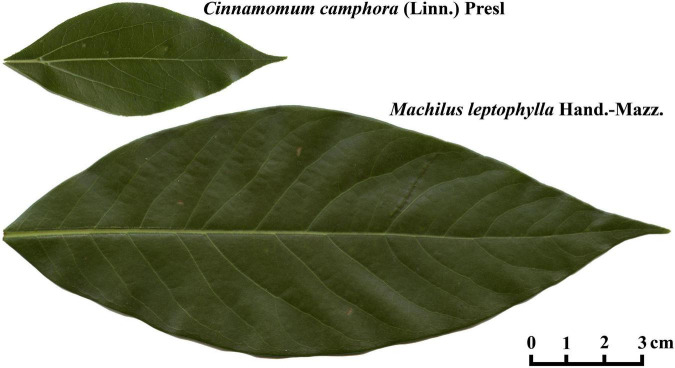
Leaf examples of *Cinnamomum camphora* (L.) Presl and *Machilus leptophylla* Hand.-Mazz. (Lauraceae).

Regardless of the increase of support cost, the larger the leaf size, the greater the photosynthetic returns. However, there is a trade off between leaf size and support cost (Milla and Reich, 2007; Niklas et al., 2007). In addition, increases in leaf size also require increases in the investment of the leaf petiole (Niklas, 1991). The bilateral symmetry of leaf shape is helpful to reduce the cost of development at the earlier stage of leaf formation and also matches the evolution of the leaf venation system (Smith et al., 1997; Runions et al., 2017; Kierzkowski et al., 2019). Thus, it is valuable to explore the association of leaf size and structure with the corresponding functions. In this study, we use two Lauraceae species, *Cinnamomum camphora* (CC) and *Machilus leptophylla* (ML), both of which have an ovate leaf shape (Figure 2), to test: (i) whether the MLRF equation is valid for describing the leaves of the two species, (ii) whether leaf shape (represented by the lamina centroid ratio) can affect the allocation of mass between leaf petiole and lamina, and (iii) whether the extent of lamina bilateral asymmetry can affect the validity of the MLRF equation.

## Materials and Methods

### Leaf Collection and Measurement

From two Lauraceae species, *Cinnamomum camphora* (L.) Presl, and *Machilus leptophylla* Hand.-Mazz., which will be referred to as CC and ML for convenience, more than 600 leaves were sampled from three trees for each species from early- to mid-August 2020 at the Nanjing Forestry University Campus (32°07′59″N, 118°81′37″E) and the Nanjing Botanical Garden of the Chinese Academy of Sciences (32°05′12″N, 118°83′47″E). Based on 100 randomly sampled leaves for each species, the ratio of leaf petiole length to lamina length is 0.26 ± 0.04 for CC, and 0.14 ± 0.02 for ML; the diameter of the leaf petiole is 0.15 ± 0.02 cm for CC, and 0.28 ± 0.03 cm for ML. On average, leaf petiole length is ca. 1/4 of lamina length for CC, and ca. 1/7 of lamina length for ML; CC has a longer and slenderer petiole than ML. Mature and intact leaves with leaf petioles were randomly sampled from the middle canopy between 8 am and 9 am. To reduce water loss, the sampled leaves were put into plastic self-sealing bags (28 cm × 20 cm), and quickly brought back to the laboratory at the Nanjing Forestry University Campus to measure lamina mass and leaf petiole mass, the total time lapse being less than two hours from the collection sites to the laboratory.

Lamina mass and leaf petiole mass were measured using an electronic balance (ME204/02, Mettler Toledo Company, Greifensee, Switzerland; measurement accuracy 0.0001 g), and the lamina image was scanned with an Epson scanner (V550, Epson Indonesia, Batam, Indonesia) at 600 dpi resolution. The images were converted to black–white images and saved as bitmap images at a 600 dpi resolution by Adobe Photoshop (version: 13.0). Then, the MATLAB (version ≥ 2009a) procedure developed by Shi et al. (2018b) was used to extract the planar coordinates of the leaf edges, and the R (based on R version 3.6.1; R Core Team, 2019) script proposed by Su et al. (2019) was used to measure lamina length, width, and area.

### Statistical Methods

Lobry et al. (1991) proposed a model (i.e., LRF equation) to describe the effect of temperature on the growth rate of microbial populations. Shi et al. (2017) modified the LRF equation (i.e., MLRF equation) by adding a parameter δ to improve the fitting elasticity and used its integral to develop a new sigmoid growth equation. After adjusting the curves generated by the MLRF equation to make them more bilaterally symmetrical along the *x*-axis, we found that the following equation validly described ovate leaf shapes of many plants (Shi et al., 2021):


$$y=y_{c}\,{\left[\frac{\left(x-x_{1}\right)\,{\left(x-x_{2}\right)}^{2}}{\left(x_{2}-x_{c}\right)\,\left[\left(x_{2}-x_{c}\right)\,\left(x-x_{c}\right)-\left(x_{1}-x_{c}\right)\,\left(x_{c}+x_{2}-2\,x\right)\right]}\right]}^{\delta },$$

where, *x*_1_ and *x*_2_ represent, respectively, the lamina base as the starting point and the lamina apex as the ending point, *x*_c_ represents the point on the lamina length axis associated with lamina maximum width, *y*_c_ represents half lamina maximum width, and δ is a parameter influencing the curvature of the curve. The curvature of the lamina edge can be directly represented by the parameter δ of the ovate leaf shape model. A large δ value signifies a large curvature for the lamina edge [see Figure 1C of Shi et al. (2021)]. This equation produces half an ovate leaf shape, with the other half generated by *f*(*x*) = −*y*. In order to estimate the parameters of the MLRF equation, the Nelder-Mead optimization (Nelder and Mead, 1965) method was used to minimize the residual sum of squares (RSS):


$$\text{RSS}=\sum_{j=1}^{n}{\left(y_{j}-{\hat{y}}_{j}\right)}^{2},$$

where, *n* represents the number of data points on the lamina edge, the subscript *j* represents the *j*th point, and *y*_*j*_ with a circumflex represents the fitted response variable. For comparing the goodness of fit of the model to the lamina edge data of the two species, we calculated the adjusted root-mean-square error (RMSE_adj_) of each lamina (Wei et al., 2019; Shi et al., 2020b):


$${\text{RMSE}}_{\text{adj}}=\frac{\sqrt{\text{RSS}\,/\,n}}{\sqrt{A\,/\,\pi }},$$

where, *A* is the lamina area. This indicator accounts for the proportion of the mean absolute deviation in the *y* values to the radius of an assumed circle whose area equals lamina area.

**FIGURE 3:**
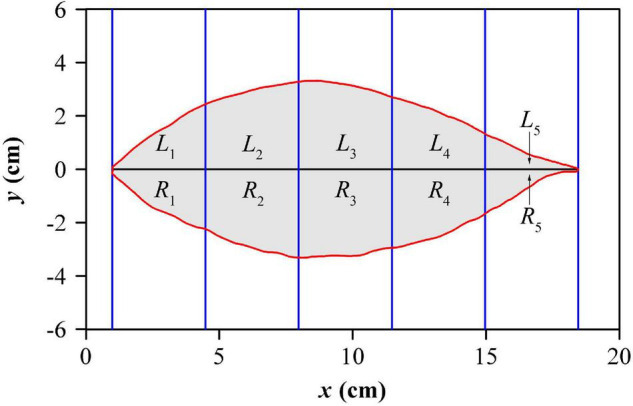
Illustration for lamina bilateral asymmetry measure. For clarity, five equidistant strips are exhibited here, but 1000 strips were used in the actual calculation.


To measure the extent of lamina bilateral asymmetry, the standardized index (SI) proposed by Shi et al. (2018a) was calculated for each leaf. The indicator took a certain number of equidistant strips (rectangles) to intersect with a leaf, as shown in Figure 3. To obtain a more accurate value, we actually used 1,000 strips, but to conveniently show this approach only five strips were used in this figure. In each strip, the intersection between the strip and the lamina was divided into upper and lower parts, and their areas were represented by *L*_*i*_ and *R*_*i*_ respectively, where, *i* represents the *i*th strip. The mathematical expression of SI is as follows:

$$\text{SI}=\frac{1}{1000}\,\sum_{i=1}^{1000}\frac{\left|L\,i-R\,i\right|}{L\,i+R\,i}.$$

The smaller the SI, the smaller the degree of the bilateral asymmetry of a lamina. A prior study showed the log-transformation of SI made its distribution more normal (Shi et al., 2020a). Thus, we used the natural logarithm, i.e., ln SI, in the interspecific comparison.

To compare the significance of the difference in the extent of lamina bilateral asymmetry between the two species studied, leaf shape (reflected by the ratio of lamina width to lamina length, and the ratio of *x*_c_ to *x*_2_), and the ratio of leaf petiole mass to lamina mass, the analysis of variance was carried out at the 0.05 significance level. The Pearson correlation coefficient test was used to test the significance of the correlation between the ratio of leaf petiole mass to lamina mass and the centroid ratio, and the correlation between the goodness of fit using the leaf shape model (reflected by RMSE_adj_) and the extent of lamina bilateral asymmetry. All statistical analyses were performed using R (version 3.6.1) (R Core Team, 2019).

## Results

**FIGURE 4:**
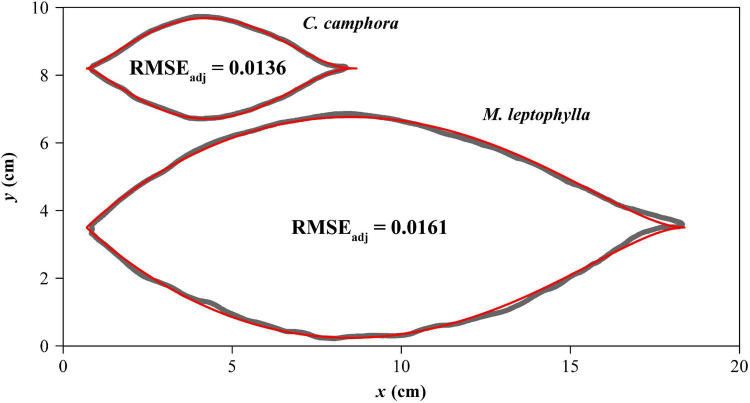
Comparisons between the scanned leaf edges (gray curves) and the leaf edges predicted by the MLRF equation (red curves) for one leaf example each of *Cinnamomum camphora* (L.) Presl and *Machilus leptophylla* Hand.-Mazz. The adjusted root-mean-square error (RMSE_adj_) is used to reflect the goodness of fit.

**FIGURE 5:**
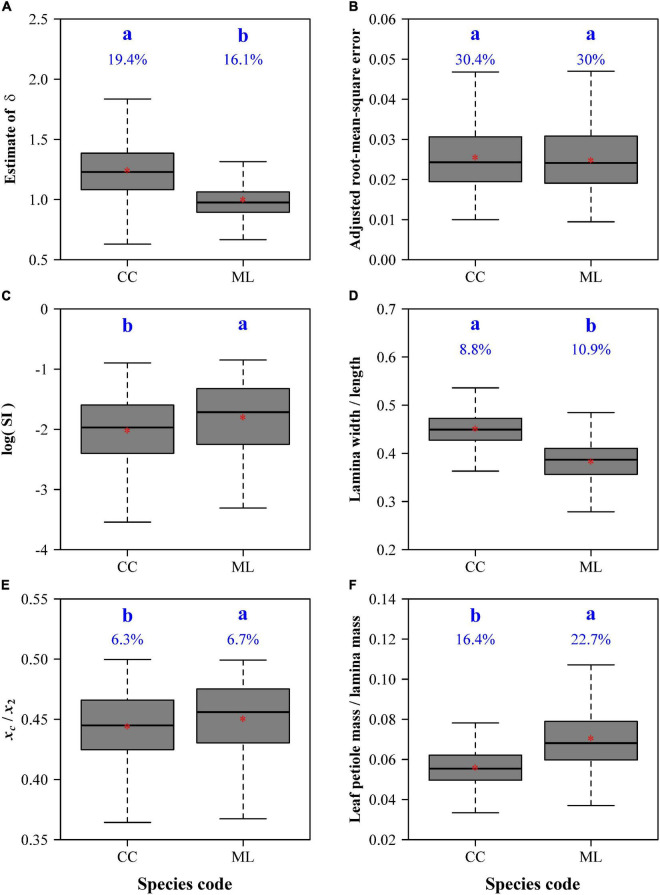
Comparisons of the estimates of δ **(A)**, adjusted root-mean-square errors **(B)**, the natural logarithms of the standardized indices for lamina bilateral asymmetry **(C)**, ratios of lamina width to length **(D)**, centroid ratios (i.e., *x*_c_/*x*_2_) **(E)**, and ratios of leaf petiole mass to lamina mass **(F)** between *C. camphora* (represented by CC) and *M. leptophylla* (represented by ML). In each panel, the letters a and b are used to represent the significance of the differences between the two species, with species sharing a common letter not differing significantly at the 0.05 significance level; the percentage numbers at the top of the whiskers represent the coefficients of variation; the horizontal lines in boxes represent medians, and the asterisks in boxes represent means.

The adjusted RMSEs of the MLRF equation for all laminas were smaller than 0.05, which verifies the validity of the MLRF equation in describing the ovate leaf shapes studied here. This shows that the mean absolute deviation between the observed and predicted *y* values is less than 5% of the radius of an assumed circle whose area equals lamina area for each of the 616 leaves. Figure 4 exhibits two leaf examples and the predicted leaf shapes using the MLRF equation. Whether it is necessary to introduce a parameter to control the curvature in the MLRF equation was answered here; Figure 5A showed that most estimates of δ for CC were larger than 1, and the mean estimated δ of CC was significantly larger that of ML. This means that the leaf shape of CC has a larger curvature than that of ML. However, there was no significant difference in the goodness of fit between the two species according to the calculated adjusted RMSE values (Figure 5B). See Supplementary Table 1 for details.

There were significant differences in leaf bilateral asymmetry (Figure 5C), the ratio of lamina width to length (Figure 5D), the ratio of leaf petiole mass to lamina mass (Figure 5E), and the centroid ratio (Figure 5F). CC has a more bilaterally symmetrical and broader leaf shape than ML; the lamina centroids are both close to the lamina bases for the two species, but the centroid of CC is closer to the lamina base; the ratio of leaf petiole mass to lamina mass of CC is significantly smaller than that of ML.

There was a significant negative correlation between the centroid ratio and that of leaf petiole mass to lamina mass for CC (*r* = –0.14; *P* < 0.05), but a significant positive correlation for ML (*r* = 0.30; *P* < 0.05). For the pooled data, a significant positive correlation was found (*r* = 0.17; *P* < 0.05).

**FIGURE 6:**
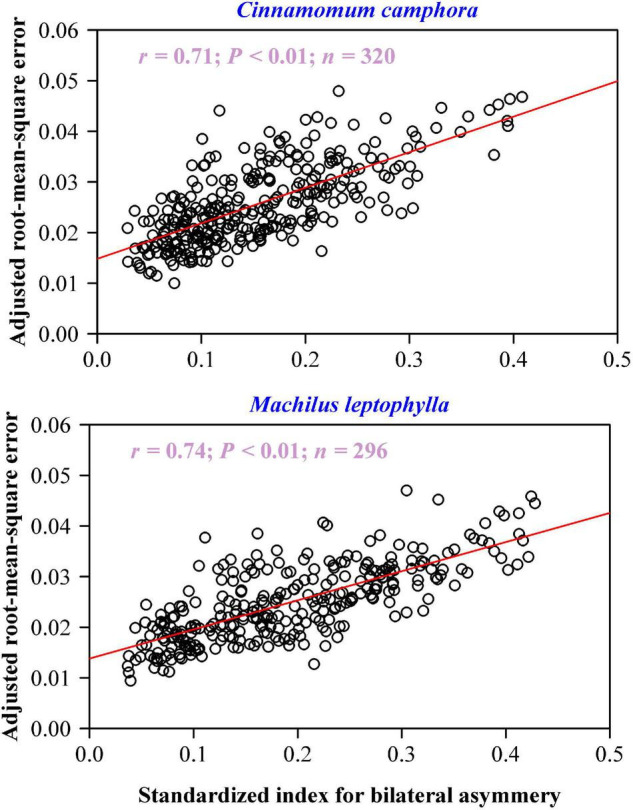
Correlation between the adjusted root-mean-square error and standardized index for lamina bilateral asymmetry. In each panel, the open circles represent the observations; the red straight line represents the regression line; *r* is the coefficient of correlation; *n* is the sample size.

There was a significant positive correlation between the adjusted RMSE values and the SI values for each species: the coefficient of correlation of *C. camphora* was 0.71 (*P* < 0.01), and that of *M. leptophylla* was 0.74 (*P* < 0.01) (Figure 6), which means that approximately 50% of the unexplained variation using the MLRF equation can be further accounted for by the extent of lamina bilateral asymmetry.

## Discussion

### Link of the Validity of the Leaf Shape Equation to Lamina Bilateral Asymmetry

Although the extant leaf shape models (Dornbusch et al., 2011; Shi et al., 2015; Li et al., 2021) have been verified to be valid for plants with special leaf shapes, the question of lamina bilateral asymmetry has been little considered. The current study shows that the MLRF model has explained more than 95% of the mean absolute deviation in *y* values relative to the radius of an assumed circle whose area equals lamina area. The remaining prediction errors could be explained by the extent of leaf bilateral asymmetry, and the explained variation approximated 50%. This work implies that the validity of a leaf shape model is closely related to the extent of leaf bilateral asymmetry. For a leaf shape apparently deviating from a bilateral asymmetry hypothesis, it is necessary to introduce one or more parameter(s) to a model for reflecting such a deviation (Huang et al., 2020; Li et al., 2021). However, in the current study, the leaf shapes of two Lauraceae species are basically bilaterally symmetrical, and there is only a slight deviation in lamina apex from the lamina length axis (i.e., the symmetrical axis). This is a small but important functional modification for leaves to conveniently drain the water on the leaf surface (Wang et al., 2020). In future studies on developing other leaf shape models, investigators will have to consider whether it is necessary to introduce one or more parameter(s) to reflect the lamina asymmetry by checking whether the deviation from a perfect bilateral symmetry is large or negligible. Through the correlation analysis on RMSE_adj_ and SI, we found that the degree of lamina asymmetry significantly influenced the goodness of fit of the model. The larger the degree of lamina asymmetry, the larger the RMSE_adj_. Thus, the degree of lamina asymmetry can be quantified by the goodness of fit of the MLRF equation, which suggests that similar approaches are promising in quantifying or evaluating the degree of lamina bilateral asymmetry in future studies related to leaf asymmetry.

Apparently, the leaf shape characteristics including lamina bilateral symmetry or asymmetry are a result of the interactions for plants and environmental factors (Chitwood and Sinha, 2016). Plants growing in different environments show corresponding structural and physiological adaptabilities to external environmental conditions, such as moisture and carbon dioxide content (Ito et al., 2015; de Boer et al., 2016); light capture efficiency and the location of the leaves in the crown can both influence leaf shape (Reich et al., 1998). The leaf bilateral symmetry of CC was better than ML. The following reasons might have led to this result. For one thing, the lamina size of ML is larger, and it requires a more skewed lamina apex to rapidly drain the water on the leaf surface (Wang et al., 2020). Also, the ratio of lamina width to length of ML is significantly smaller than that of CC (Figure 5D), and the narrow leaf shape tends to cause large variations in the symmetrical distributions of hierarchical reticulate veins and in the cell division rates on both sides of the lamina (Dengler and Kang, 2001).

### Influences of the Lamina Centroid Ratio and Leaf Petiole Pattern on the Investment of Mass to Leaf Petiole

The tradeoff between leaf photosynthetic investment and leaf support investment is always a study hotspot in botany (Niklas, 1991; Niinemets et al., 2007). Previous studies show that lamina mass positively correlates with lamina area, and leaf petiole mass also positively correlates with lamina mass on a log-log scale (Niklas, 1991; Li et al., 2008). Leaf shape has been demonstrated to affect the scaling relationship between lamina mass and lamina area (Lin et al., 2020), but few studies have been carried out to examine whether leaf shape can change the scaling relationship between leaf petiole mass and lamina mass. In this work, we analyzed the correlation between the lamina centroid ratio and the ratio of leaf petiole mass to lamina mass for two Lauraceae species, and found a negative correlation for the long and slender petiole (CC) and a positive correlation for the short and thick leaf petiole (ML).

The ratios of lamina width to length of CC and ML differ significantly. CC has a smaller and broader lamina, which greatly reduces the supporting requirement for the leaf petiole. Thus, smaller and broader leaves tend to have long and slender leaf petioles and are little influenced by the centroid ratio. ML has a larger and narrower lamina that increases the burden of the leaf petiole, so leaf petiole mass correlates with lamina mass. When the lamina centroid is far away from the lamina base, the plants tend to have short but thick leaf petioles to support laminas for maintaining the maximum light surface (Takenaka, 1994). Large laminas need the petiole to enhance the ability to conduct water, but also endure the role of external forces (Niklas, 1999), which requires increasing the investment of mass to the leaf petiole. The results indicated that lamina size and shape can significantly modulate the allocation of investment between the lamina mass and leaf petiole mass, thereby affecting leaf development patterns in different environments.

Fluctuating asymmetry is widely used as a measure of developmental stability (Palmer and Strobeck, 1986; Palmer, 1994). It regards the difference in a trait of interest between two sides of a sample as a developmental “noise.” There are many indices to measure the level of fluctuating asymmetry, and the SI can be deemed as one indicator of fluctuating asymmetry [compared with FA2 of Palmer (1994)]. This is to say, the indicator here measured the extent of leaf fluctuating asymmetry. It is somewhat valuable to compare different fluctuating asymmetry indices for recommending the best one. However, the present work mainly focuses on: (i) the description of the ovate leaf shape using a known parametric model, i.e., the MLRF model, based on a large sample size, and (ii) whether the centroid ratio, which is obtained from the parameters of the ovate leaf shape model, can have a significant influence on the allocation of biomass to the petiole and lamina. Although we quantified the extent of lamina bilateral asymmetry using SI, it was used only to explore whether the goodness of fit of the ovate leaf shape model is associated with the extent of lamina bilateral asymmetry. The results showed that lamina bilateral asymmetry (which reflects SI) accounted for ca. 50% of the unexplained variation of the MLRF model. Our ovate leaf shape model actually hypothesizes (predicts) a perfectly bilateral symmetrical leaf shape, i.e., an ideal norm without developmental instability, so any existing asymmetry for laminas will more or less weaken the model’s power. Fortunately, given developmental stability of leaves, the studied laminas are of nice bilateral symmetry except a minor deviation (which we can regard as a ‘modification’ from a bilateral symmetry) at the lamina apex for a functional drainage requirement. It is necessary to point out that fluctuating asymmetry is a population parameter rather than a sample parameter (Graham, 2021), so it requires using a large sample size to reflect an accurate asymmetrical trait of interest. To serve our study aim of testing whether the validity of the MLRF model is related to the extent of lamina bilateral asymmetry based on individual leaves (samples), for the two studied species, 320 and 296 leaves were used respectively, which should represent the general traits of lamina structure including lamina bilateral asymmetry.

## Conclusion

In this study, we tested the validity of the MLRF model for describing leaf shapes of two species of Lauraceae using a large sample for each species. The equation was confirmed to be credible to describe the actual shapes, and all adjusted root-mean-square errors (RMSEs) were smaller than 0.05. This means that the mean absolute deviation is smaller than 5% of the radius of an assumed circle whose area is equal to the lamina area. We also found that the goodness of fit of the MLRF model relied on the extent of lamina bilateral asymmetry. The prediction error can be further accounted for by the lamina bilateral asymmetry, and it explained ca. 50% of the unexplained variation of the MLRF model. In addition, we did not find consistent evidence that the centroid ratio is positively correlated with the ratio of leaf petiole mass to lamina mass, which is perhaps associated with leaf petiole patterns. A long and slender leaf petiole tends to correspond to a small and broad leaf, regardless of the centroid ratio; a short and thick leaf petiole tends to correspond to a large and narrow leaf, and the ratio of leaf petiole mass to lamina mass is more likely to positively correlate with the lamina centroid ratio. This work provides important insights into the link between leaf structure and function.

## Data Availability Statement

The original contributions presented in the study are included in the article/Supplementary Material, further inquiries can be directed to the corresponding authors.

## Author Contributions

DR, HW, and PS designed the work, analyzed the data, and revised the manuscript. YL and YZ carried out the experiment and wrote the initial draft. All authors commented on and agreed with this submission.

## Conflict of Interest

The authors declare that the research was conducted in the absence of any commercial or financial relationships that could be construed as a potential conflict of interest.

## Publisher’s Note

All claims expressed in this article are solely those of the authors and do not necessarily represent those of their affiliated organizations, or those of the publisher, the editors and the reviewers. Any product that may be evaluated in this article, or claim that may be made by its manufacturer, is not guaranteed or endorsed by the publisher.
